# Application of Field’s massage therapy in an extremely premature infant: Case report

**DOI:** 10.15649/cuidarte.4762

**Published:** 2026-03-25

**Authors:** Hernando Parra Reyes, Adriana Camila Rincón Ascanio, Ednna Rocío Peña Vargas, Vanessa Tatiana Galvis Pinto

**Affiliations:** 1 Hospital Internacional de Colombia, Fundación Cardiovascular de Colombia; Floridablanca, Colombia. E-mail: hernandoparra@fcv.org Hospital Internacional de Colombia Floridablanca Colombia hernandoparra@fcv.org; 2 Hospital Internacional de Colombia, Fundación Cardiovascular de Colombia; Floridablanca, Colombia. E-mail: adrianarinconascanio@fcv.org Hospital Internacional de Colombia Floridablanca Colombia adrianarinconascanio@fcv.org; 3 Hospital Internacional de Colombia, Fundación Cardiovascular de Colombia; Floridablanca, Colombia. E-mail: ednnapena@fcv.org Hospital Internacional de Colombia Floridablanca Colombia ednnapena@fcv.org; 4 Hospital Internacional de Colombia, Fundación Cardiovascular de Colombia; Floridablanca, Colombia. E-mail: vanessagalvis@fcv.org Hospital Internacional de Colombia Floridablanca Colombia vanessagalvis@fcv.org

**Keywords:** Infant, Premature, Massage, Nursing Care, Intensive Care, Neonatal, Recién Nacido Prematuro, Masaje, Atención de Enfermería, Cuidado Intensivo Neonatal, Recém-Nascido Prematuro, Massagem, Cuidados de Enfermagem, Terapia Intensiva Neonatal

## Abstract

**Introduction:**

Field’s massage therapy is a simple, practical, and cost-effective intervention for weight gain in premature infants. It has demonstrated significant benefits in increasing gastric motility, improving nutrient absorption, and reducing hospital stays. The present case report aimed to describe the nursing care plan for a premature infant who received Field’s massage therapy for weight gain over five days, using standardized nursing language and nursing disciplinary conceptual-theoretical support.

**Case description:**

An extremely premature female infant, born at 25.2 weeks of gestational age with low birth weight and multiple comorbidities, underwent Field’s massage therapy as the primary intervention for weight gain. The nursing diagnosis identified was Imbalanced nutrition: Less than body requirements [00002], related to prematurity. The nursing intervention on which the application of Field’s massage therapy was based was Massage [1480], and the outcome evaluated was Weight: Body mass [1006]. After five days of the intervention, the patient exhibited a 9.6% increase in weight compared to baseline, with an average daily weight gain of 17.5 g/kg/day.

**Conclusion:**

Field’s massage therapy proved to be an effective nursing intervention to promote weight gain in a premature infant hospitalized in an intensive care unit. The therapy contributed to positive clinical outcomes through an approach of humanization and patient-centered care.

## Resumen

**Introducción:**

La terapia de masaje de Field es una intervención sencilla, práctica y costo-efectiva para la ganancia de peso en prematuros. Esta terapia ha demostrado importantes beneficios para el aumento de la motilidad gástrica, una mejor absorción de nutrientes y la reducción de las estancias hospitalarias. El presente informe de caso tiene como objetivo describir el plan de atención de enfermería para una paciente prematura que recibió terapia de masaje de Field para la ganancia de peso durante cinco días, a través del uso del lenguaje estandarizado de enfermería y el sustento conceptual-teórico disciplinar.

**Descripción del caso:**

Paciente recién nacida extremadamente prematura de 25,2 semanas de edad gestacional, con bajo peso al nacer y múltiples comorbilidades a quien se le aplicó la terapia de masaje de Field como intervención principal. El diagnóstico de enfermería identificado fue Desequilibrio Nutricional: Menos que los requerimientos del cuerpo [00002] relacionado con prematuridad. La intervención de enfermería desde la cual se sustentó la aplicación de la terapia de masaje de Field fue Masaje [1480] y el resultado evaluado fue Peso: masa corporal [1006]. Cinco días después de la intervención, la paciente mostró un aumento del 9,6% con respecto al peso inicial, con un promedio de aumento diario de 17,5gr/kg/día.

**Conclusiones:**

La terapia de masaje de Field demostró ser una intervención de enfermería efectiva para la ganancia de peso de una paciente prematura hospitalizada en una unidad de cuidado intensivo. La terapía contribuyó a obtener resultados clínicos positivos desde un enfoque de humanización y atención centrada en el paciente.

## Resumo

**Introdução:**

A terapia de massagem de campo é uma intervenção prática, simples e econômica para ganho de peso em recém-nascidos prematuros. Ela demonstrou benefícios significativos no aumento da motilidade gástrica, levando a uma maior absorção de nutrientes e à redução do tempo de internação hospitalar. Este artigo descreve o plano de cuidados de enfermagem para um paciente prematuro, utilizando a técnica de massagem de campo para ganho de peso ao longo de um período de cinco dias, utilizando linguagem padronizada de enfermagem e uma base conceitual e teórica disciplinar.

**Descrição do Caso:**

Este relato apresenta um recém-nascido prematuro extremo de 25,2 semanas de idade gestacional, com baixo peso ao nascer e múltiplas comorbidades. A intervenção primária foi a massagem com a terapia de massagem de campo para ganho de peso. O diagnóstico de enfermagem presente no paciente foi desequilíbrio nutricional: ingestão abaixo das necessidades relacionada à prematuridade [00002], a intervenção da qual se baseou a aplicação da terapia de campo foi a massagem [1480] e o resultado avaliado foi o ganho de peso [1006] onde após a aplicação da intervenção foi evidenciado um aumento de 9,6% sobre o peso inicial após 5 dias, com aumento médio diário de 17,5gr/kg/dia.

**Conclusões:**

A aplicação da massagem de campo demonstrou ser uma intervenção de enfermagem eficaz para ganho de peso em pacientes prematuros em ambientes de terapia intensiva; contribuindo assim para desfechos clínicos positivos a partir de uma abordagem de humanização e cuidado centrado no paciente.

## Introduction

According to the World Health Organization (WHO), approximately one in ten children worldwide is born prematurely, and this is considered a major global public health concern. In 2020, the incidence of preterm births ranged from 4% to 16%, corresponding to an estimated 13.4 million infants born before 37 weeks of gestation, one of the leading causes of mortality among children under five years of age^1^. At the national level, data from the National Administrative Department of Statistics of Colombia (DANE) reported a prevalence of preterm births of 11% in 2022, 0.3 percentage points above the data reported in 2021 (10.7%)^2^. At the local level, within the Cardiovascular Institute of the Hospital Internacional de Colombia (HIC), a total of 484 births were attended in 2023, of which 41.73% (n=202) were preterm newborns, and 37.39% (n=181) required specialized care in the neonatal intensive care unit (NICU)^3^.

In NICUs, cutaneous stimulation received in the intrauterine environment, through contact with amniotic fluid and the uterine walls, is interrupted as a result of premature birth. The minimal handling required and the lack of contact with parents, due to the infant’s critical health status, may negatively impact growth and neurodevelopment^4^. Accordingly, nursing proposes the implementation of comprehensive interventions aimed at promoting weight gain in premature infants^5^. These interventions are focused on somatic stimulation that contributes to enhancing vagal tone and gastric motility, leading to more efficient nutrient absorption and improved weight gain. Kinesthetic stimulation has also been associated with bone growth by increasing bone-specific alkaline phosphatase levels, which increase bone volume^6^.

Field’s massage therapy involving moderate pressure is one of the somatic and kinesthetic stimulation interventions that, when applied to premature infants, has shown significant beneficial effects and clinically relevant outcomes, such as weight gain and stimulation, enhancing neurodevelopment. These effects have also been linked to the reduction in the length of hospital stay in intensive care units for these patients^7^.

Based on moderate-pressure stroking, Field’s massage therapy has demonstrated positive effects, such as reduced behavioral signs of stress, increased alertness, more organized motor activity, and increased postnatal growth. Some studies have documented significantly greater weight gain in preterm infants receiving massage therapy, particularly when both tactile and kinesthetic components are included, following the protocol developed by Tiffany Field^8^.

Implementing this type of intervention in premature infants in NICUs enables nurses to organize and plan the neonatal care process in a way that leads to clinical outcomes that contribute to shorter hospital stays.

This study aims to document the weight gain of a premature infant receiving Field’s massage therapy through a nursing care plan that applies standardized nursing language and integrates conceptual, theoretical, and disciplinary foundations guided by Sister Callista Roy’s Adaptation Model of Nursing and Kristen Swanson’s Theory of Caring.

## Case description

The case of an extremely premature infant of 25.2 weeks of gestational age is presented, with an appropriate birth weight for her age of 710 g (50th/90th percentile) and a height of 34 cm. Maternal background: 33-year-old mother; obstetric history: 2 pregnancies, 1 delivery, 1 cesarean section, 2 live births (G2P1C1V2). The cesarean delivery was due to chorioamnionitis treated in other institution. At birth, the newborn requires guided neonatal adaptation, advanced ventilatory support with orotracheal intubation, and administration of two doses of pulmonary surfactant. The patient has multiple comorbidities: neonatal seizures, newborn respiratory distress syndrome, bronchopulmonary dysplasia, prolonged mechanical ventilation, patent ductus arteriosus (PDA) with three failed pharmacological closures, and, finally, surgical closure and neonatal sepsis requiring multiple courses of antibiotic therapy.

Admission to the NICU occurred at one month of postnatal life (40 days) and a corrected gestational age of 31.2 weeks. She weighed 840 g and measured 37 cm, indicating low weight for gestational age. Treatment begins with Field’s massage therapy at 36.5 weeks of corrected gestational age (80 days postnatal). Her total hospital stay was 3 months and 12 days. The patient was enrolled during her stay in the NICU, with prior informed consent provided by her natural guardian (mother).

**Nursing assessment**

The nursing assessment conducted on the newborn followed the systematic framework of the nursing care process based on Sister Callista Roy’s Adaptation Model. This approach enables the identification of ineffective adaptive responses caused by each type of stimulus across the four adaptative modes: physical-physiological, role function, interdependence, and self-concept-group identity. Likewise, from the theoretical approach that underpins the institutional nursing care model (Kristen Swanson’s Theory of Caring), the categories or concepts of doing for, being with, and enabling are brought to life in the intervention and outcome stages, standardized through the use of the Nursing Outcomes Classification (NOC) and Nursing Interventions Classification (NIC) taxonomies.

**Stimuli assessment**

**Focal stimuli:** Decreased weight gain

**Contextual stimuli:** Prematurity.

**Residual stimuli: **Urban area origin

**Physical-physiological mode**

**- Basic functions**

**1. Oxygenation needs:** Newborn female with guided neonatal adaptation (Apgar score of 6/10 at 1 minute and 7/10 at 5 minutes) with positive pressure ventilation. She required orotracheal intubation after delivery and immediate administration of exogenous pulmonary surfactant, with a second dose administered two days after birth. At the referring institution, she was diagnosed with patent ductus arteriosus (PDA) with hemodynamic significance, for which three pharmacological closure attempts were made, initially with paracetamol, followed by ibuprofen, without successful closure. She was subsequently transferred to our institution for surgical closure of the PDA and continuation of comprehensive management of prematurity Upon admission to the institution, the newborn had prolonged mechanical ventilation, radiographs indicated marked changes consistent with bronchopulmonary dysplasia, and echocardiography confirmed hemodynamically significant PDA. Physical examination revealed rhythmic heart sounds, a grade IV/VI murmur, palpable peripheral pulses, and adequate distal perfusion. The patient required a packed red blood cell transfusion and initiation of vasodilator therapy with milrinone. Subsequently, surgical closure of the ductus arteriosus was performed, followed by progressive discontinuation of vasodilator support and gradual reduction in ventilatory support. The patient also required methylxanthine therapy for the management of apneic episodes.

At the time of assessment, prior to initiation of the intervention, the newborn was receiving oxygen via a conventional nasal cannula at 0.1 L/min. She exhibited mild substernal retractions, adequate respiratory rate and oxygen saturation, and symmetrical breath sounds. Hemodynamically, she presents regular heart sounds, no murmurs, normal capillary refill, and palpable peripheral pulses, with mean arterial pressure within the normal range and heart rate with occasional tachycardia (157-183 beats per minute [bpm]). At that time, she did not require methylxanthine therapy.

**Ineffective responses:** Poor neonatal adaptation, pulmonary surfactant deficiency, need for prolonged mechanical ventilation, grade 1 bronchopulmonary dysplasia, sustained need for oxygen delivered via conventional nasal cannula, inability to tolerate oxygen weaning, substernal retractions, hemodynamically significant PDA, grade IV/VI murmur, anemia, and tachycardia.

**2. Nutrition needs:** At birth, anthropometric measurements of the neonate were as follows: weight 710 g, length 34 cm, head circumference 22.5 cm, and chest circumference 21.5 cm. According to the Fenton growth chart for preterm infants, the patient was classified as having appropriate weight for gestational age (50th/90th percentile). Upon admission to our institution, her anthropometric measurements were weight 840 g, length 37 cm, head circumference 24 cm, chest circumference 23.5 cm, and abdominal circumference 26 cm. Based on the Fenton growth chart, she was classified as underweight for gestational age (3rd/10th percentile). A weight gain of 130 g was observed over 40 days, corresponding to a growth velocity of 3.68 g/kg/day. She was admitted from the referring facility, receiving parenteral nutrition support and enteral stimulation with 5 ml of breast milk (47.6 cc/kg/day).

On admission, physical examination revealed a soft, distended, and compressible abdomen, nontender to palpation; however, oral intake was withheld due to her clinical history, with adequate blood glucose levels maintained. Supportive care was initiated with maintenance intravenous fluids, followed by total parenteral nutrition. After the surgical procedure, and once the patient was more stable, enteral feeding was initiated using breast milk and preterm formula (24 kcal/oz), with a gradual increase in volume and no reported complications. Between 40 and 80 days of life, the patient gained 1345 g, corresponding to a growth velocity of 15.38 g/kg/day.

The procedure began on the newborn girl, who was 80 days old, 36.5 weeks of corrected gestational age, and weighed 2185 g. She was receiving full enteral feeding with preterm formula (24 kcal/oz), administered as 42 mL every three hours, for a total of eight feedings per day, providing a caloric intake of 123 kcal/kg/day. Feeding was achieved by a combination of suckling and gavage. She also received vitamin supplementation and occasional kangaroo mother care due to the mother’s psychosocial factors.

**Ineffective responses:** Upon admission to the institution, the newborn was classified as underweight for gestational age. During the first 40 days, a weight gain of 130 grams was observed, equivalent to a growth rate of 3.68 g/kg/day.

**3. Elimination needs:** The newborn demonstrated adequate renal function throughout her hospital course, both at the referring institution and upon admission to our institution, with blood urea nitrogen of 6.95 mg/dL and serum creatinine of 0.37 mg/dL. Urine output was within normal limits, with no edema and no signs of fluid overload.

**4. Activity-rest needs:** The newborn had experienced sleep disruption since birth due to the interventions performed by nurses, physicians, and other interdisciplinary healthcare staff required for her comprehensive care.

**Ineffective responses:** Difficulty initiating sleep due to repeated interruptions during handling and joint manipulations by the care team.

**5. Protection needs:** Maternal history included serologic tests for toxoplasmosis and rubella, six prenatal checkups, and three normal prenatal ultrasounds. The mother developed chorioamnionitis, characterized by leukocytosis and a hyperthermic uterine cavity. Given this history, early-onset neonatal sepsis was suspected at birth, and first-line antibiotic therapy was initiated. During her initial hospitalization at the referring institution, the neonate developed ventilator-associated pneumonia (VAP) and late-onset neonatal sepsis.

Upon admission, laboratory tests showed no evidence of ongoing infection. The newborn’s blood type was O+, and the non-treponemal test was non-reactive. During her stay, she received prophylactic antibiotic therapy related to the surgical closure of the PDA. At the time of assessment, the newborn had intact skin and no pressure-related injuries.

The newborn was diagnosed with stage 1 retinopathy of prematurity (ROP) in zone II without plus disease at two months of age, showing spontaneous regression and no indication for intervention.

**Ineffective responses: **Ineffective responses: Ventilator-associated pneumonia, retinopathy of prematurity, late- onset neonatal sepsis.

**- Complex functions**

**1. Senses:** Newborn without pain upon admission, comfort measures were implemented, and minimal-handling protocol was initiated due to her prematurity. Because of her underweight and poor clinical condition, the activities of the interdisciplinary team are grouped in a single intervention, which includes vital sign monitoring, diaper changes, position changes every eight hours, respiratory therapy if required, nursing and medical assessment, laboratory testing, and care of the peripherally inserted central catheter (PICC) in accordance with institutional protocols.

Following surgical closure of the ductus arteriosus, the newborn’s postoperative pain was evaluated using the CRIES scale (Crying, Requires oxygen, Increased vital signs, Expression, Sleeplessness), which yielded a score of 4/10. Analgesic management was provided with paracetamol, along with comfort measures.

At the time of assessment, the patient showed no signs of pain, exhibited adequate thermoregulation, was no longer under the minimal-handling protocol, and appeared comfortable. Comfort measures, such as containment, nesting, scheduled position changes every three hours, an d environ mental stimuli control (noise, light, and temperature) were applied.

**Ineffective response: **Postoperative pain.

**2. Fluids, electrolytes, and acid-base balance:** No umbilical cord blood gas analysis was available at birth. Upon admission to our institution, arterial blood gas results indicated compensated respiratory acidosis, with the following values: pH 7.26, PCO₂ 67.2 mmHg, PO₂ 39.4 mmHg, lactate 1.27 mmol/L, and CHCO₃ 29.9 mmol/L. The patient was started on basal intravenous fluids under a restricted fluid regimen for a total intake of 130 cc/ kg/day, and parenteral nutrition was initiated thereafter to provide metabolic support.

At the time ofassessment, priortoinitiationoftheintervention, thenewbornhadadequateenteral intake, with total fluids of 150 cc/kg/day, and no evidence of fluid or electrolyte imbalance.

**Ineffective response: **Compensated respiratory acidosis on admission.

**3. Neurological function:** At birth, the newborn with guided neonatal adaptation exhibited signs of generalized hypotonia and incomplete assessment of primitive reflexes due to extreme prematurity. She was admitted without a cranial ultrasound available from the referring facility records.

At the time of assessment, the patient presented with anterior fontanelle soft and flat, spontaneous eye opening, and symmetrical movement of all four extremities, with no abnormal movements observed. The cranial ultrasound performed at our institution through the anterior fontanelle showed normal findings, with no evidence of intraventricular hemorrhage. However, owing to recurrent respiratory pauses without an identifiable pulmonary cause, anticonvulsant therapy with phenobarbital was initiated. A three-hour video electroencephalogram (EEG) demonstrated abnormal multifocal epileptiform activity, predominantly in the left temporal region, with occasional discharges in the right occipital and left central regions. Consequently, phenobarbital therapy was maintained, and levetiracetam was added to the treatment regimen. A follow-up EEG performed at a corrected gestational age of 34.2 weeks showed normal brain electrical activity with no evidence of seizures.

**Ineffective response: **Axial hypotonia, immature primitive reflexes, multifocal epileptiform activity.

**4. Endocrine function:** Newborn screening for congenital hypothyroidism performed at birth yielded normal results. Upon admission to our institution, the patient demonstrated adequate metabolic regulation and normal blood glucose levels, with all reports within normal ranges.

**• Role function mode**

**1. Primary role: **Extremely premature female newborn at 25.2 weeks of gestational age.

**2. Secondary role: **Daughter, granddaughter, niece.

**3. Retiary role: **Not applicable to newborns.

**• Interdependence mode**

The need for healthcare personnel to fulfill and perform her physiological functions.

**• Self-concept-group identity mode**

Not applicable to newborns.

**Nursing care plan**

**NANDA-I Diagnosis: **[00002] Imbalanced nutrition: Less than body requirements. This imbalance is related to prematurity and evidenced by body weight below the expected range for age and sex and a neonatal weight gain of < 30g/day.

**Rationale:** Preterm newborns face a hostile environment for growth, which requires highly energy- demanding physiological processes for survival, such as feeding, thermoregulation, and complete maturation. The lower the gestational age, the greater the adversity of the conditions due to the immaturity of metabolic mechanisms responsible for nutrient utilization, and the higher the nutritional requirements. Therefore, extremely premature infants exhibit postnatal growth below intrauterine expectations.

Furthermore, postnatal complications (e.g., periods of fasting, respiratory failure, sepsis, or heart disease) may impair adequate nutrition or increase metabolic expenditure, thereby affecting growth. Three stages have been considered for nutrition management in premature infants: adaptation, stabilization, and growth. It is important to evaluate nutritional behavior during the stabilization stage, as this is the period when Field’s massage should be implemented. This therapy, usually applied after the first week of life, aims to achieve early recovery of birth weight and a growth rate similar to that in utero for the gestational age. Therefore, one of the fundamental goals in NICUs is to provide nutrition that ensures growth as close as possible to that expected in an intrauterine environment, estimated at 17 to 20 g/kg/day^9^.

Study results agree that various comorbidities are associated with weight loss and significantly lower weight gain in premature infants with prolonged hospitalizations, even when provided with combined parenteral and enteral nutrition, kangaroo mother care, humidified incubators, accurate estimation of insensible water loss, and appropriate intravenous fluid management^10^. This is why strategies to promote weight gain in preterm newborns with multiple comorbidities need to be implemented, thereby reducing the prolonged hospital stays of these patients.

In accordance with the above, implementing activities based on Kristen Swanson’s Theory of Caring is essential, particularly the concepts of doing for and being with, integrated with the Nursing Intervention Classification (NIC) and informed by the results of the case report on Field’s massage therapy.

*Doing for* represents a fundamental form of caring that involves performing for another what they would do for themselves if able. Doing for involves comforting, anticipating, and protecting others, addressing their needs skillfully and competently to promote patients’ well-being and respect their dignity^10^.

**NOC:** [1006] Weight: Body mass

**Definition:** Extent to which body weight, muscle, and fat are congruent to height, body frame, gender, and age.

**Indicators:**

[100601] Weight

[100609] Weight percentile (Fenton, girls)

This outcome highlights the being with category from Kristen Swanson’s Theory of Caring, which refers to being present for the person and includes being there in person, showing availability, and sharing feelings without overwhelming the person. It involves the nursing staff’s authentic presence for the patients, who are cared for through emotional presence. Being with someone is a way of sharing the meanings, feelings, and experiences of the person being cared for^10^.

**NIC: **[1240] Weight gain assistance

**Definition:** Facilitating gain of body weight

**Activities:**

Weigh patient at specified intervals, as appropriate.

Chart weight gain process

**NIC: **[1480] Massage

**Definition:** Stimulation of the skin and underlying tissues with varying degrees of hand pressure to decrease pain, produce relaxation, and/or improve circulation.

**Activities:**

Establish a period of time for a massage that achieves the desired response.

Place in a comfortable position that facilitates massage.

Adapt the massage area, technique, and pressure to the purpose of the massage.

Evaluate and document response to massage.

The intervention was initiated when the newborn reached a corrected gestational age of 36.5 weeks and a weight of 2185 g, based on criteria of enteral intake greater than 50.00%, absence of signs of infection, and hemodynamic stability. The procedure was performed by two nurses in the presence of the mother, who was instructed in the massage technique for later application. The therapy consisted of Field’s massage, which combines moderate pressure and kinesthetic stimulation, without gloves or oils, for 15 minutes, divided into 5 minutes of massage, 5 minutes of kinesthetic stimulation, and finally 5 minutes of massage again. The routine was repeated three times daily for five consecutive days. Table 1 describes the massage proposed by Tiffany Field^6^.

**Table 1:** Field’s massage therapy

Phase	Step	Procedure
1. Moderate pressure, prone position	1 2 3 4 5	With the palm of the hand held flat, from the head to the neck and back again. *Duration 1 minute* From the neck across the shoulders and back again. *Duration 1 minute* From the upper back to the waist and back again. *Duration 1 minute* From the hip to the foot to the hip on both legs. *Duration 1 minute* From the shoulder to the hand and back to the shoulder on both arms. *Duration 1 minute*
2. Kinesthetic stimula- tion, supine position	1 2 3 4 5	Flexion and extension of the right upper limb, 6 repetitions. *Duration: 1 minute, 10 seconds apiece for each flexion.* Flexion and extension of the left upper limb, 6 repetitions. *Duration: 1 minute, 10 seconds apiece for each flexion.* Flexion and extension of the lower right limb, 6 repetitions. *Duration: 1 minute, 10 seconds apiece for each flexion.* Flexion and extension of the lower left limb, 6 repetitions. *Duration: 1 minute, 10 seconds apiece for each flexion.* Flexion and extension of both lower limbs simultaneously, 6 re- petitions. *Duration: 1 minute, 10 seconds apiece for each flexion.*
3. Moderate pressure, prone position	Repeat again phase 1	

During the five consecutive days of the intervention, the newborn was weighed daily at 8:00 a.m. to monitor and document weight gain, fluid and caloric intake, vital signs, and signs of stress at each session.

To evaluate stress during each intervention, three types of responses were used as indicators: autonomic (A), motor (M), and state (E) responses:

**Autonomic responses:**

1. Alteration in respiratory rate and/or breathing pattern.

2. Alteration in skin color.

3. Alteration in heart rate.

4. Decrease in oxygen saturation.

5. None.

**Motor responses:**

1. Avoidance of eye contact or turning the head away from the stimulus.

2. Frowning.

3. Torsional movements of arms, legs, or trunk.

4. Exaggerated and sustained extension of arms and/or legs.

5. None.

**State responses:**

1. Mouth opening or yawning.

2. Eye movement without establishing eye contact.

3. Nausea

4. Sneezing and/or hiccupping.

5. None.

Vital signs (heart rate, respiratory rate, oxygen saturation, and skin temperature) were also monitored throughout the intervention to assess autonomic responses. The variables were operationalized as follows:

**Heart rate:**

1. < 110 (bpm)

2. 110 to 180 bpm.

3. > 180 bpm

**Respiratory rate:**

1. < 40 breaths per minute.

2. 40 to 60 breaths per minute.

3. > 60 breaths per minute.

**Oxygen saturation:**

1. ≤ 90%

2. > 90%

**Skin temperature:**

1. < 36.5°C

2. 36.5 to 37.5°C

3. > 37.5°C

All data collected in this study are freely available for access and consultation on Mendeley Data^11^.

**Ethical considerations**

This work was submitted to the Scientific and Research Ethics Committee of the Fundación Cardiovascular de Colombia and was approved for dissemination purposes (Approval code RC- 2024076).

Informed consent was obtained from the patient’s mother.

## Results

**Figure 1:**
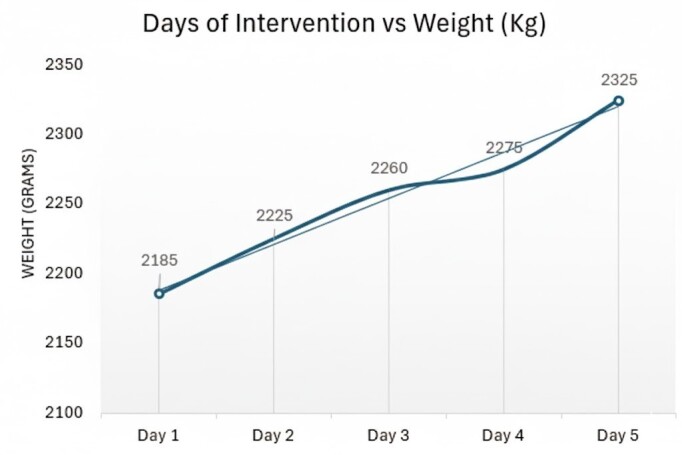
Daily weight gain during the intervention

The intervention was conducted over five consecutive days, with each phase lasting five minutes. Daily weight measurements were documented. Figure 1 illustrates the daily weight gain, showing the initial weight of 2185 g and a progressive daily increase, with no decrease at any point of the intervention. By the final day, the newborn’s weight reached 2395 g, representing a gain of 210 g, equivalent to a 9.61% increase from the initial weight and a growth rate of 17.53 g/kg/day. According to the Fenton growth chart, this value corresponds to an adequate weight for gestational age (50th percentile). The intervention thus demonstrated a weight gain consistent with that expected for premature neonates hospitalized in a NICU^9^. Furthermore, the growth velocity during the intervention (17.53 g/kg/day) exceeded that recorded during the 40 days preceding the intervention (15.38 g/kg/day).

**Figure 2:**
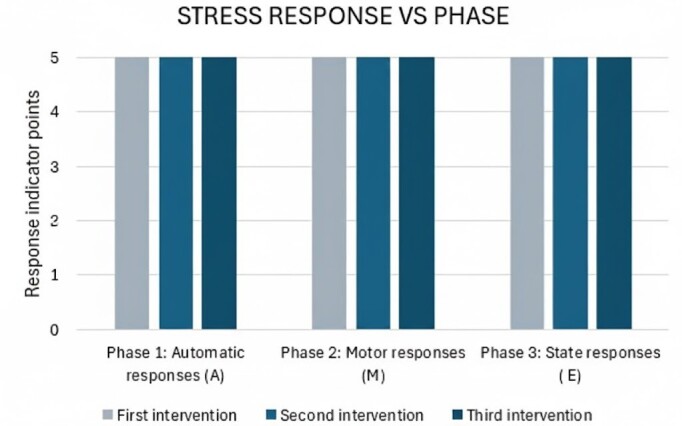
Stress responses in the intervention phases

Simultaneous monitoring of vital signs (heart rate, respiratory rate, oxygen saturation, and skin temperature) revealed an adequate stress response. No autonomic, motor, or state responses indicative of stress were observed, as shown in Figure 2.

Furthermore, studies have been found in the literature reporting the positive influence of somatic stimulation on brain development in premature newborns, possibly mediated by the action of insulin-like growth factor 1 (IGF-1) as an underlying mechanism. These studies also indicate that when a massage protocol is applied, the extrauterine brain maturation of low-risk premature infants resembles the intrauterine developmental trajectory that would have occurred had intrauterine gestation continued. Additional benefits documented for massage therapy in hospitalized premature infants include a reduced risk of neonatal sepsis, shorter hospital stays, and lower stress levels^4^.

## Conclusions

According to the nursing care plan developed for the premature patient, effective weight gain can be achieved through the application of Field’s massage therapy. The importance of providing holistic care grounded in conceptual–theoretical frameworks such as Sister Callista Roy’s Adaptation Model is highlighted, which in turn facilitates the identification of the need to intervene following Kristen Swanson’s Theory of Caring, whose concepts guide the process of nursing activities.

Field’s massage therapy with moderate pressure and kinesthetic stimulation is proposed as a nursing intervention for the comprehensive management of premature infants. This therapy supports physiological adaptation, particularly in relation to nutritional function, and may contribute to shorter hospital stays in the Neonatal Intensive Care Unit (NICU).

Field’s massage therapy enables nurses not only to provide holistic care but also to strengthen the nurse-patient relationship through sustained physical contact with the newborn and recognize each newborn’s unique needs to address them, contributing to their adaptation to the extrauterine and hospital environment.

Through the proposed intervention, family-centered and individualized care is provided, facilitating the care of premature infants and involving them in activities that contribute to their overall well- being.

## References

[1] Organización Mundial de la Salud (2023 ). Nacimientos prematuros.

[2] Departamento Administrativo Nacional de Estadística (Jun 2023 ). Principales resultados de estadísticas vitales de nacimientos y defunciones para el primer trimestre de 2023, el año acumulado 2022 y el año corrido 2023.

[3] Estadísticas cesáreas y partos atendidos en el Instituto Cardiovascular del Hospital Internacional de Colombia Archivo estadística ICV-HIC.

[4] Álvarez  MJ, Rodríguez-González  D, Rosón  M, Lapeña  S, Gómez-Salgado  J, Fernández-García D ( 2019). Efectos de la masoterapia y la kinesiterapia para desarrollar la antropometría del recién nacido prematuro hospitalizado: un estudio cuasi-experimental. J Pediatr Nurs.

[5] Lu  LC, Lan  SH , Hsieh   YP, Lin  LY , Chen   JC, Lan  SJ  (2020 ). Massage therapy for weight gain in preterm neonates: A systematic review and meta-analysis of randomized controlled trials. Complement Ther Clin Pract.

[6]  Field   T ( 2014). Massage therapy research review. Complement Ther Clin Pract.

[7] Field   T, Diego   M,  Hernandez-Reif   M ( 2010). Preterm infant massage therapy research: a review. Infant Behav Dev.

[8]  Acevedo-Olguín   AL,  Iglesias-Leboreiro  J,  Bernárdez-Zapata   I,  González-Morán   RJ, Rendón- Macías   ME (2018 ). Crecimiento ponderal intrahospitalario en pretérminos de peso adecuado y bajo al nacimiento. Rev Mex Pediatr.

[9] Mena  P,  Milad  M,  Vernal  P ,  Escalante MJ  (2016 ). Nutrición intrahospitalaria del prematuro. Recomendaciones de la Rama de Neonatología de la Sociedad Chilena de Pediatría. Rev Chil Pediatr.

[10] Raile Alligood,   M (2022). Nursing Theorists and their work. ElSevier.

[11]  Parra Reyes   HR, Ascanio   AC,  Peña Vargas   ER, Galvis Pinto   VT “Aplicación técnica field a un neonato prematuro extremo para un reporte de caso”. Mendeley Data, V1.

